# Effectiveness of beta-tricalcium phosphate in comparison with other materials in treating periodontal infra-bony defects around natural teeth: a systematic review and meta-analysis

**DOI:** 10.1186/s12903-021-01570-8

**Published:** 2021-04-29

**Authors:** Reham AL Jasser, Abdulelah AlSubaie, Fayez AlShehri

**Affiliations:** 1 Associate Professor,Department of Periodontics and Community Dentistry, Dental College, King Saud University, PO Box 60169, Riyadh, 11545 Saudi Arabia; 2 Saudi Board Resident, Ministry of Health, Riyadh, Saudi Arabia

**Keywords:** Beta-tricalcium phosphate, Intra-bony defect, Periodontal surgery, Regeneration, Bone fill, Pocket depth reduction, Clinical attachment gain

## Abstract

**Background:**

Beta-tricalcium phosphate in regenerative surgery has shown promising results in terms of bone gain and new vital bone formation; however, several studies have contradicted this finding. The aim of this study was to evaluate the effectiveness of beta-tricalcium phosphate compared to other grafting materials in the regeneration of periodontal infra-bony defects.

**Methods:**

Electronic database (Cochrane, MEDLINE, PubMed, Embase, Science Citation Index Expanded) and manual searches for related data were performed up until March 2020. The outcomes were pocket depth reduction, clinical attachment level gain, and amount of bone fill.

**Results:**

Five studies were selected based on the inclusion criteria. Bone regeneration with beta-tricalcium phosphate was observed to be superior to that with debridement alone but showed comparable results to other bone graft materials in terms of pocket depth reduction, clinical attachment level gain, and bone fill. Regenerative procedures for periodontal infra-bony defects that used beta-tricalcium phosphate in combination with other growth factors yielded superior outcomes. The meta-analysis revealed that for cases with two-wall defects, the use of beta-tricalcium phosphate yielded statistically significant differences in pocket depth reduction and clinical attachment level gain, but not in bone fill.

**Conclusions:**

Beta-tricalcium phosphate appears to be a promising material for use in periodontal infra-bony defect regeneration around natural teeth. However, randomized clinical trials with larger sample sizes and more controlled study designs are needed to support these findings.

## Background

Tricalcium phosphate is found as four chemically polymorphs: α, super-α, γ, and β [1]. Beta-tricalcium phosphate (β-TCP) was introduced in 1973 by Driskell [2] as a material to treat bone defects caused by trauma. β-TCP is a bioceramic material used in the medical and dental field [3]. Animal studies have proven its usefulness in several dental procedures, such as pulp capping and apexification in endodontics [4], repair of cleft palates and orbital rim defects in maxillofacial surgery [5, 6], and repair of osseous lesions in periodontics [7]. β-TCP is a biocompatible [4, 6, 8] alloplastic bone grafting material that is resorbable with osteoconductive properties [9]. Therefore, it is considered as a good alternative to autografts or allografts for certain grafting procedures. It has been shown to undergo complete resorption and is replaced by bone within a period of 0.5–1.5 years [10] when applied to various bony defects, such as intraosseous defects around natural teeth, edentulous defective alveolar ridges, and maxillary sinuses [9, 11].

The amount of mature bone that this material can provide in a grafted area is crucial. When grafted sites were histologically evaluated, β-TCP particles were observed to be surrounded by and are in intimate contact with osteoid. Furthermore, fragments of mature bone appeared separate from the synthetic material with minimal traces of inflammation. This suggests that β-TCP undergoes complete resorption and replacement by mature bone. However, this process might take years to complete [12]. Several attempts have been made to measure the amount of mature bone formation with this grafting material over different periods of time. A histological study evaluating the re-osteointegration process of a bony defect around implants in the area of the distal surface of the first molar and the mesial surface of the second molar showed a bone gain of 1.90 mm and 1.41 mm around the first and second molars, respectively [13].

Kishore T et al. reported a mean bone fill (BF) of 3.6 mm and 4.4 mm with β-TCP alone after 6 and 9 months, respectively [14]. As such, this grafting material has shown promising results in grafting procedures, compared to the gold standard autografts and allografts. Saini et al. conducted a split-mouth design study comparing β-TCP alone and in combination with platelet-rich plasma and observed linear BF with β-TCP alone [15]. When β-TCP was combined with the membrane, the BF was 3.9 mm and 4.2 mm after 6 and 9 months, respectively.

Several studies utilizing β-TCP alone or in combination with other grafting materials in several different surgical regenerative procedures have shown promising results for BF and new vital bone formation, in a manner that is comparable to that achieved with other bone grafting materials such as allografts and xenografts [16–18]. In contrast, some studies have contradicted previous statements. A study by Snyder et al. reported an inferior outcome with β-TCP, compared with other grafting materials [11]. Thus, the literature shows conflicting results and there is a lack of information regarding the exact amount of BF associated with different surgical regenerative procedures when using β-TCP. Therefore, the aim of this systematic review was to evaluate the use of β-TCP alone and combined with other substitutes for bone regeneration around natural teeth.

### Focused question

What are the periodontal regenerative outcomes when using β-TCP as a grafting material (whether alone or in combination with growth factors), and how are they different from those with other grafting materials when used in guided tissue regeneration (GTR) of infra-bony defects around natural teeth?

## Methods

### Study design

We conducted a systematic review of studies focusing on the use of β-TCP in combination with other bone graft materials for the regeneration of bone defects around natural teeth. As currently recommended, we followed the PRISMA Statement checklist for reporting a systematic review [19].

### Registration

The protocol for this systematic review was specified in advance and registered with the International Prospective Register of Systematic Reviews (PROSPERO) on 6/1/2020.

### Eligibility criteria for study inclusion

To conduct the systematic review, we assessed all studies in which the primary objective was to evaluate the benefit of β-TCP combined with other bone grafts in GTR around natural teeth. Randomized clinical trials, case series, and case reports were eligible for inclusion. Thereafter, the eligibility criteria (by applying the PICO framework) were as follows:

- Population: Patients with periodontal infra-bony defects (including 1, 2, or 3 walls) around natural teeth.
- Intervention: Graft material composed mainly of β-TCP.
- Control: All other graft materials used to treat such defects.

Outcomes:

- Primary: pocket depth reduction (PD reduction), clinical attachment level gain (CAL gain), and amount of BF.
- Secondary: keratinized tissue width (KTW), gingival recession (GR), and soft tissue thickness change (ΔSTT).

### Search strategy

A comprehensive three-step search strategy was established to identify studies for this systematic review. No language restrictions were applied. Electronic searches of the MEDLINE (via PubMed), EMBASE, and Cochrane, Science Citation Index Expanded databases and manual searches of unpublished data, academic theses, and journals were conducted up until March 2020. Additionally, the reference lists and trial registries were searched, and regulatory agency websites and manufacturers were queried. This search and subsequent review took a period of 6 months. The online database search was performed using the following search strategy prepared for MEDLINE: (((((Periodontal regeneration) OR infra bony defects) OR furcation defects) OR guided tissue regeneration) OR guided bone regeneration) OR bone augmentation))))) AND ((((((((bone fill) OR periodontal pocket) OR clinical attachment level) OR keratinized tissue) OR bone regeneration) OR soft tissue regeneration) OR recession) OR furcation fill)))))))) ((Tri calcium phosphate) OR calcium phosphate) OR synthograft).

### Selection of included studies

Two independent reviewers (A.S and F.S) screened the titles, abstracts, and full texts of the papers that were identified. Disagreements between the reviewers were resolved through discussion until consensus was reached. Inter-reviewer agreement for the selection process was assessed using Cohen’s Kappa score [20]. The reasons for excluding studies were recorded. Studies meeting the inclusion criteria underwent data extraction and synthesis.

### Data extraction

A pre-designed form was developed to extract the following data: Author name(s); publication year and place; source of funding; conflict of interest; study design; sample size; follow-up period; source, selection, and description of the study population (including age, sex, race, ethnicity, and presence and characteristics of GR at baseline); definition and measurement method of the intervention; controls; outcomes; results and their variations; and risk-of-bias.

### Data synthesis

The data were organized into evidence tables according to PRISMA guidelines [19], and a descriptive summary was created to determine the study’s characteristics, quality, and results. Descriptive statistical analysis according to the mean values was used to evaluate the outcomes (Table 1).

**Table 1:** Qualitative description of the included studies

Name of author	Country	Population	Intervention	Follow-up	Comparison	Outcomes
Strub et al. 1979	Switzerland	*Patients* 8 *Age* 28 − 55 years *Gender* 5 M, 3F *Bony defects* 47 *Defect Type* 1-, 2-, 3-Wall defects or horizontal bone loss *Pre-surgical Preparation* OHI* SRP* Occlusal adjustment Splinting Re-evaluation after 4–6 weeks *Antibiotic use* 4 million IU oral penicillin 1 day pre-surgery	TCP* *Form* TCP was mixed with sterile distilled water (38.5% powder to 61.5% water) to form a paste	12 months	Frozen allogenic graft	*Primary outcomes* PD* reduction (TCP): 1.8 mm (allograft): 2.0 mm Re-entry BF* (TCP): 1.2 mm (Allograft): 1.5 mm *Secondary outcomes* *Radiographic BF* (TCP): 1.05 mm (Allograft): 0.9 mm *Residual pocket deeper than 3* *mm (TCP)* 38% (Allograft): 22%
Snyder AJ et al., 1984	USA	*Patients* 10 *Age* Unknown *Gender* Unknown *Bony defects* 10 *Defect type* 1- or 2-wall, furcation areas *Pre-surgical preparation* Initial-phase therapy Occlusal analysis *Antibiotics* Tetracycline 250 mg tablets, q.i.d 10 days post-surgery	TCP *Form* Die-pressed to form discs 2 inches in diameter × 1/8-inch thick and fired at 2000°F for 2 h. The discs were then crushed in an alumina mortar and pestle, with the resulting powder being sieved to recover the 200/ + 325 mesh size fraction	18 months	None	*Primary outcomes* *PD reduction* 3.6 mm CAL* gain 1.2 mm *Re-entry BF* 2.8 mm
Zefiropoulos GG et al., 2007	Germany	*Patients* 64 *Age* 30 − 71 years *Gender* 31 M, 34 F *Smoking status* 28 S*, 37 NS* *Bony defects* 93 *Defect type* 2 or 3 walls *Pre-surgical Preparation* Non-surgical therapy Re-evaluation *Antibiotics* Diclofenac 100 mg per day for 4 days, started 1 day pre-surgery	HA/b-TCP + ASB *	12 months	ASB* ASB + BDX*	*Primary outcomes* *CAL gain* (HA/b-TCP + ASB): 3.2 mm (ASB): 3.4 mm (BDX): 3.2 mm *Re-entry BF* (HA/b-TCP + ASB): 1.6 mm (ASB): 2.8 mm (BDX): 1.5 mm *Secondary outcomes* BOP* reduction (HA/b-TCP + ASB): 13.8% (ASB): 14.7% (BDX): 20.0% PLI*reduction (HA/b-TCP + ASB): 27.6% (ASB): 26.5% (BDX): 30.0% RBG*percentage (HA/b-TCP + ASB): 82.3% (ASB): 69.3% (BDX): 83.3%
Rajesh JB et al., 2009	India	*Patients* 60 *Age* 20 − 45 years *Gender* Not mentioned *Bony defects* 60 *Defect types* 2 or 3 walls *Pre-surgical preparation* OHI* SRP* Occlusal adjustment Re-evaluation after 4 weeks *Antibiotics* Doxycycline 100 mg, BID for the 1st day followed by 100 mg OD for 5 days	CPC *Form* Chitra Calcium Phosphate Cement in the form cement	12 months	Debridement only (Deb) Hydroxyapatite cement granules (HA)	*Primary outcomes* *PD reduction* (CPC): 6.20 mm (HA): 4.05 mm (Deb): 2.95 mm *CAL gain* (CPC): 5.80 mm (HA): 3.55 mm (Deb): 2.30 mm *Secondary outcome* GR* reduction (CPC): 0.15 mm (HA): 0.15 mm (Deb): 0.20 mm
Sukumar S et al., 2010	Czech Republic	*Patients* 21 *Age* 21–53 years *Gender* 8 M, 13 F *Smoking status* 7 S, 14 NS *Bony defects* 39 *Defect Types* 2 or 3 walls *Pre-surgical preparation* OHI* SRP* Elimination of local factors Occlusal adjustment Re-evaluation after 2 weeks *Antibiotics* Amoxicillin 250 mg with clavulanic acid 125 mg or clarithromycin 500 mg) were prescribed to the patients for 7–14 days	TCP/CaSO_4_* *Form* Composite material consisting of beta-tricalcium phosphate + calcium sulfate	12 months	None	*Primary outcomes* *PD reduction* (TCP/CaSO_4_): 1.98 mm *CAL gain* (TCP/CaSO_4_): 1.68 mm *Secondary outcomes* GR reduction: (TCP/CaSO_4_): 0.31 mm

OHI: oral hygiene instruction; SRP: scaling and root planing; TCP: tricalcium phosphate; PD: pocket depth; BF: bone fill; CAL: clinical attachment level; S: smoker; NS: non-smoker; HA: hydroxyapatite; ASB: autogenous spongiosa; BDX: bovine-derived xenograft; BOP: bleeding on probing; PLI: plaque index by Silness and Loe; CPC: cetyl pyridinium chloride; GR: gingival recession; CaSO_4_: calcium sulfate

### Quality and risk-of-bias assessment

The methodological quality of the included studies was assessed and recorded into tables according to the PRISMA guidelines, focusing on the following points: (1) Method of randomization (e.g., the method used to generate the randomization sequence): (i) adequate, when random-number tables, a tossed coin, or shuffled cards were used; (ii) inadequate, when other methods were used, such as alternate assignment, hospital number, or odd/even date of birth; and (iii) unclear, when the method of randomization was not reported or explained. (2) Allocation concealment (e.g., how the randomization sequence was concealed from the examiners): (i) adequate, when examiners were kept unaware of the randomization sequence (e.g., using central randomization or opaque envelopes); (ii) inadequate, when other methods were used, such as alternate assignment or hospital number; and (iii) unclear, when the method was not reported or explained. (3) The blindness of examiners with regard to the treatment procedures used in the study period was assessed. (4) The completion of follow-up was based on the following question: Was the number of subjects at baseline and after the follow-up period reported? Additional assessments included the presence of explanations (reasons) for dropouts. Studies that did not report the completion of follow-up were excluded. (5) The similarity between groups at baseline. (6) Assessment of any analysis performed to control for confounding factors that can affect the final outcomes (Table 2). The risk-of-bias was graded as low, high, or unclear for each domain, based on the criteria defined in the Cochrane Handbook for Systematic Reviews of Interventions version 5.1.0 (Higgins and Green, 2011).

**Table 2:** Risk-of-bias assessment of the included studies

Authors/year	Randomization	Blinding	Incomplete outcome data	Selective outcome reporting	Similarity of groups at baseline	Control of confounding and interaction	Conflict of interest
Strub JR et al., 1979	No	No	No	No	Yes, split-mouth design	No	None
Snyder AJ et al., 1984	No	No	No	No	N/A	No	None
Zefiropoulos GG et al., 2007	No	Yes, single blinding	Yes, CAL gain	No	No	No	None
Rajesh JB et al., 2009	Yes, random-number table method	No	No	No	No	No	Yes, study was supported by the graft material company*
Sukumar S et al., 2010	No	No	No	No	N/A	No	None

*CAL* clinical attachment level

*Sree Chitra Tirunal Institute for Medical Sciences and Technology

### Quantitative analysis

Meta-analyses were performed for the three variables (PD reduction, CAL gain, and BF). As these variables are quantitative (continuous), the mean and standard deviation were used to describe them. The standardized mean difference (SMD) was used as a summary pooled statistic, where pooled effects of 0.2, 0.5, and 0.8 represented small, medium, and large effects, respectively. Student’s t-test for a single sample was used to determine the statistical significance of SMD. Cochran’s Q was used to identify the heterogeneity in the pooled data, and I^2^ values were used to observe the percentage of total variation across the studies included in the meta-analysis. A cut-off I^2^ value > 50% was used to rule out the higher levels of unexplained variability in the effect sizes. Pooled estimates were obtained using the fixed-effect and random-effect models. A *p* value of ≤ 0.05 and 95% confidence intervals were used to report the statistical significance and precision of the estimates. Graphical representation of results is shown using forest plots (overall effect using both fixed- and random-effect models) for the studies included in the meta-analysis. The analysis was performed using MedCalc for Windows version 15.0 (MedCalc Software, Ostend, Belgium).

## Results

### Reviewers’ agreement and kappa score

**Fig. 1:**
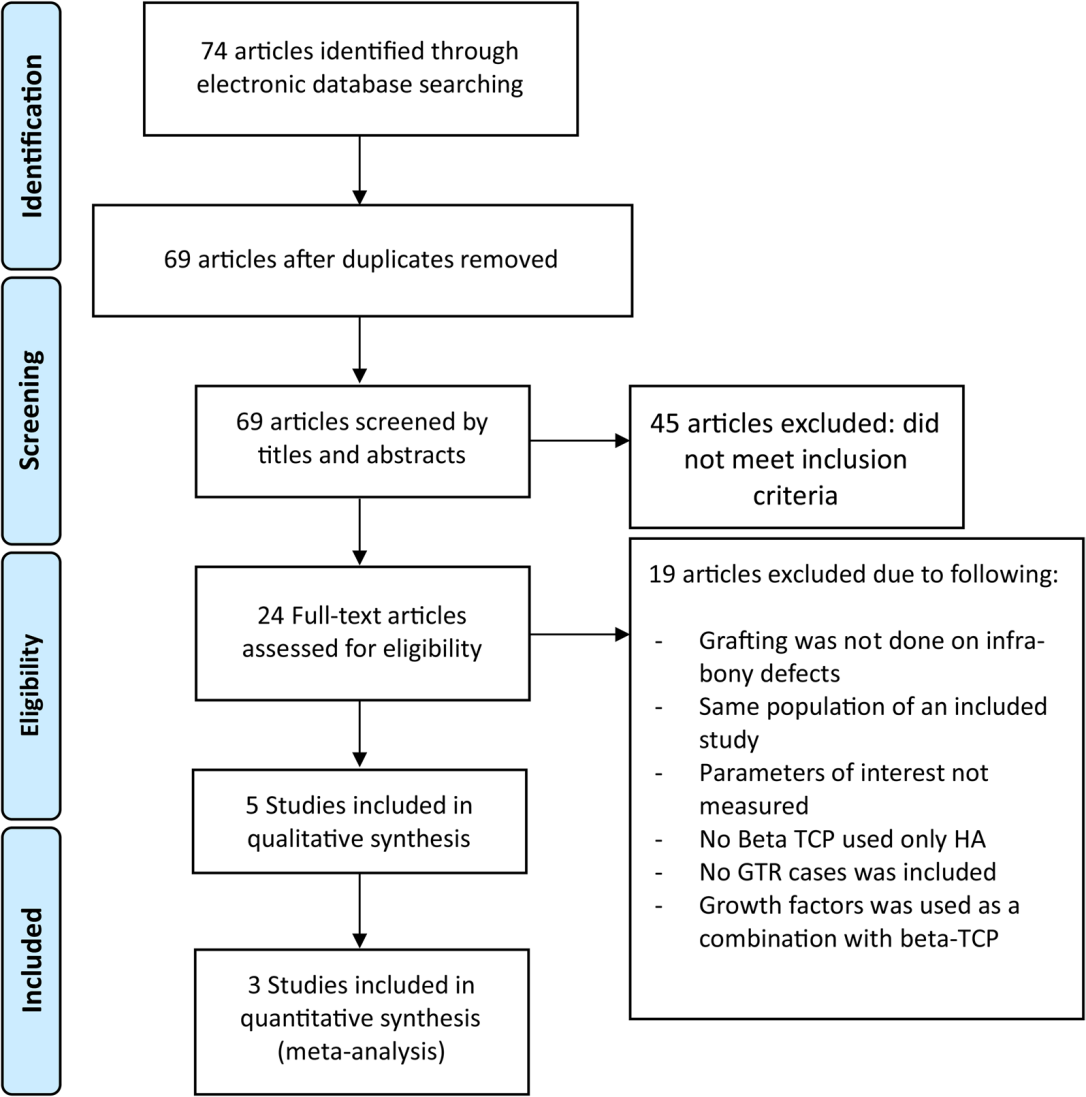
PRISMA flow diagram

Electronic searches yielded 74 articles, of which 10 were selected for full-text evaluation after screening their titles and abstracts. Five articles were further excluded, and the reasons for exclusion are listed in Fig. 1. The k value for inter-reviewer agreement for potentially relevant articles was 0.91 for full-text article reviewing, indicating an “almost perfect” agreement between the two reviewers (Fig. 1).

### Study design and patient features

Five studies were included, as shown in Table 1. The studies were published between 1979 and 2010 in the following countries: Switzerland, USA, German, India, and the Czech Republic. Four were prospective studies [11, 21–23], while one was retrospective [24]. One study used a split-mouth design to compare β-TCP versus allografts [21]. The total number of participants included in the five studies was 171 [11, 21–24]. The participants’ ages ranged from 20 to 71 years. The total number of treated sites was 254. The types of bone defects included 1-, 2-, and 3-wall defects. While patient sex was not specified in two studies [11, 23], the remaining three studies reported a total of 50 females and 44 males [21, 22, 24]. The smoking status of participants was not defined in three studies [11, 21, 23]. The study by Zafiropoulos et al. [22] defined smoking status by categorizing participants who smoked 10 cigarettes or more as a smoker, and those who smoked less than 10 cigarettes as non-smokers. The number of smokers in that study was 28, while the number of non-smokers was 37. However, they did not consider smoking status during data analysis, and the two groups were pooled together, justifying that the total sample size and the number of smokers were small. [22]. The study by Sukumar et al. mentioned that 7 participants were medium smokers; however, they did not provide a clear definition of the smoking status. The follow-up period was 12 months in three studies, 18 months in one study, and not applicable in one study [24].

### Pre-surgical preparation

The study by Strub et al. considered the initial-phase therapy, which consisted of oral hygiene instructions, scaling and root planing, occlusal adjustment, splinting, and re-evaluation after 4–6 weeks [21]. The study by Snyder et al. involved initial-phase therapy and occlusal analysis [11]. The trial by Zafiropoulos et al. included initial-phase therapy and re-evaluation [22]. Furthermore, in the study by Rajesh et al., oral hygiene instructions, scaling and root planing, occlusal adjustment, and re-evaluation after 4 weeks were performed [23]. Similarly, the study by Sukumar et al. described oral hygiene instructions, scaling and root planing, elimination of local factors, occlusal adjustment, and re-evaluation after 2 weeks [24].

### Types of interventions

Strub et al. used β-TCP mixed with sterile distilled water in a ratio of 38.5% powder to 61.5% water, yielding a paste form [21]. Snyder et al. converted TCP to powder form by a specific preparation protocol [11]. Zafiropoulos et al. used biphasic calcium phosphate (a mixture of 60% hydroxyapatite (HA) and 40% β-TCP) mixed with autogenous spongiosa (ASB) bone graft in the particulate form [22]. Rajesh et al. utilized Chitra calcium phosphate cement as an intervention in a putty form [23]. In the study by Sukumar et al., the intervention group received β-TCP with calcium sulfate, and the material was placed in a putty form (based on the manufacturer’s instructions) [24].

### Comparison groups

Three studies compared different types of bone grafts used to fill the defects. Strub et al. compared TCP powder to frozen allogenic bone [21]. Zafiropoulos et al. compared ASB alone, ASB combined with HA/β-TCP, or ASB combined with bovine-derived xenograft (BDX) [22]. Finally, Rajesh et al. compared calcium phosphate cement with HA cement and used debridement alone as a control group [23].

### Surgical approach

In the study by Strub et al., a small palatal full-thickness flap was raised, followed by granulation tissue removal from the bony defect with root planing. Bleeding was induced in the defect area followed by placement of the β-TCP/frozen allogenic graft [21]. In the trial conducted by Snyder et al., an internal bevel incision with a buccal and lingual full-thickness flap was raised. Bone defects were debrided with root planing. Then, intra-marrow penetration was performed, and tricalcium phosphate cement was grafted [11]. Zafiropoulos et al. performed an intrasulcular incision with a full-thickness flap along with a vertical incision when needed. Granulation tissue was removed, and root planing was performed. Root surfaces adjacent to the defect were conditioned with tetracycline suspension (100 mg/mL). All autogenous bone graft materials were harvested from the retromolar area. ASP alone, ASP mixed with BDX, or ASP mixed with synthetic composite (β-TCP + HA) were placed. The augmented areas were covered with a collagen membrane [22]. Rajesh et al. performed an intrasulcular incision with a full-thickness flap, debrided the defect areas, and performed root planing. Root surfaces adjacent to the defect were conditioned with tetracycline suspension (100 mg/mL) and bone graft materials were placed. [23]. Finally, Sukumar et al. performed a crevicular incision with a facial and lingual full-thickness flap and vertical incision as needed, followed by root debridement and granulation tissue removal. Root surface conditioning was performed using 2.5% tetracycline hydrochloride, and TCP/calcium sulfate was packed into the defects [24].

### Antibiotic use

Among the studies that used pre-surgical antibiotic protocols, Strub et al. prescribed penicillin 4 million IU orally 1 day before surgery [21], while Zafiropoulos et al. (2007) prescribed antibiotics with 0.1% chlorhexidine mouthwash 1 day before the surgery. [22]. Strub et al. used 0.2% chlorhexidine mouthwash twice a day for 2 weeks [21]; Snyder et al. administered tetracycline tablets 250 mg four times a day for 10 days [11]; Zafiropoulos et al. used 0.1% chlorhexidine mouthwash two times a day for 3 weeks [22]; Rajesh et al. administered doxycycline 100 mg two times a day for the first day followed by 100 mg once a day for 5 days, with 0.2% chlorhexidine mouthwash [23]; and Sukumar et al. prescribed augmentin 375 mg or clarithromycin 500 mg for 7–14 days, followed by application of hydrogen peroxide 3% during suture removal after 2 weeks and Listerine mouthwash for 2 weeks [24].

### Post-operative management

In the trial conducted by Strub et al., periodontal dressing and cyanoacrylate tissue adhesive were placed [21]. Snyder et al. placed only periodontal dressing [11]. Zafiropoulos et al. administered oral diclofenac 100 mg per day for 4 days [22]. Rajesh et al. placed a non-eugenol periodontal dressing for one week with ibuprofen 400 mg t.i.d. for 3 days [23].

### Risk-of-bias assessment

**Fig. 2:**
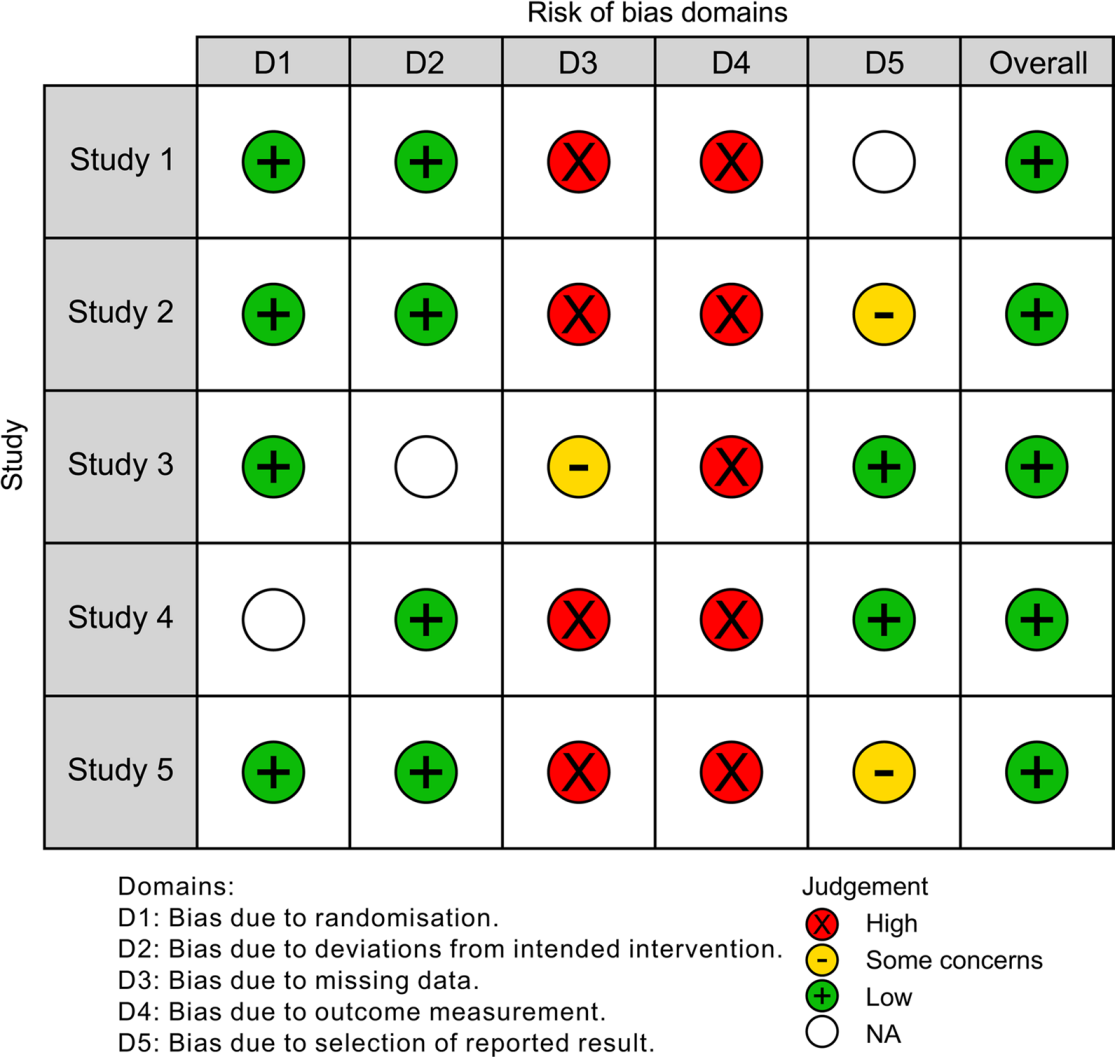
Risk-of-bias assessment (Traffic Light Plot). Overall, all the included studies showed low risk-of-bias

The results of bias assessment among the included studies are presented in Table 2. All studies obtained a low score in quality analysis (Fig. 2). Randomization and conflict of interest were reported in the study by Rajesh et al. [23]. Single blinding and incomplete outcome data were present in the study by Zafiropoulos et al. [22]. Details regarding the group similarity at baseline were not mentioned in the studies by Snyder et al. [11] and Sukumar et al. [24] (Fig. 2).

### Outcomes measured

#### Primary outcomes

##### PD reduction

Strub et al. compared frozen allogenic graft versus TCP powder and observed a net change of 2 mm for the allogenic graft, with 22% of the cases showing PD reduction greater than 3 mm; in the TCP group, the net change was 1.8 mm, with 38% of the cases showing PD reduction greater than 3 mm [21]. Snyder et al. reported a 3.6-mm PD reduction for the TCP treatment, with no group for comparison [11]. Rajesh et al. reported that PD reduction with cetyl pyridinium chloride (CPC), HA, and debridement alone was 6.2 mm, 4.05 mm, and 2.95 mm, respectively [23]. Sukumar et al. reported a PD reduction of 1.98 mm for TCP, with no comparison group [24].

##### CAL gain

Snyder et al. noted a net CAL gain of 1.2 mm for TCP, with no comparison group [11]. Zafiropoulos et al. observed that the net CAL gain for the HA/β-TCP + ASB, ASB alone, and ASB + BDX groups were 3.2, 3.4, and 3.2 mm, respectively. However, they did not compare the CAL results among the three groups [22]. Sukumar et al. reported a net CAL gain of 1.68 mm for TCP, with no comparison group [24].

##### BF

Strub et al., at re-entry, noted a 1.2-mm gain at the TCP-treated site, while the gain for the allogenic group was 1.5 mm [21]. Zaiforpoulos et al. reported a gain of 1.6 mm for HA/β-TCP + ASB, 2.8 mm for ASB alone, and 1.5 mm for ASB + BDX [22].

#### Secondary outcomes:

##### GR reduction

Rajesh et al. observed that the GR reduction was 0.15 mm for CPC, 0.15 mm for HA, and 0.2 mm for the debridement group [23]. Sukumar et al. (2010) reported a 0.31-mm increase in GR for the TCP group [24].

Meta-analysis results

#### Two-wall infra-bony defects:

**Fig. 3:**
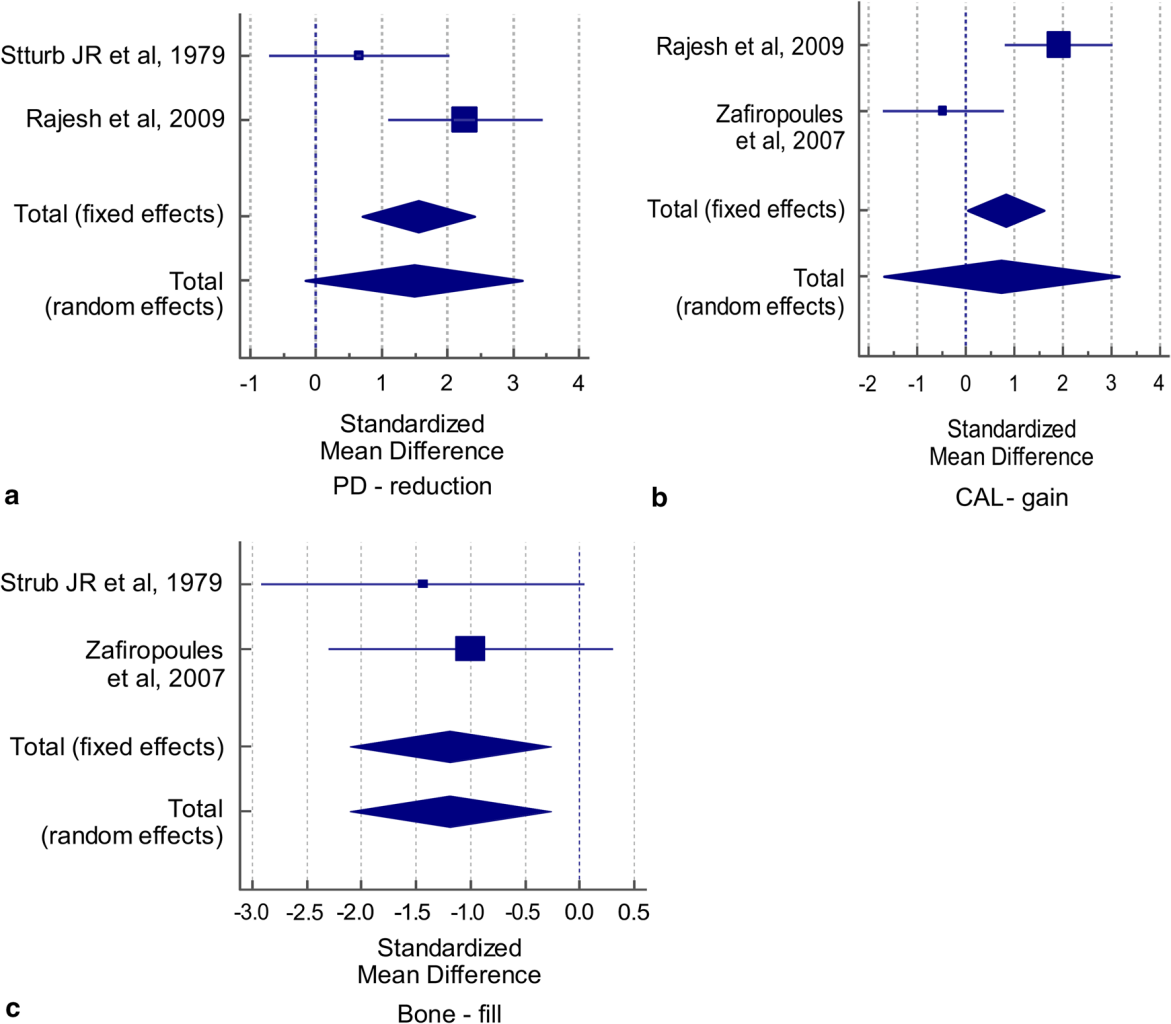
Forest plot for primary outcome variables for two-wall infra-bony defects **a** PD reduction, **b** CAL gain, **c** bone fill

For the outcome variable “PD reduction,” the statistical significance was assessed by combining the difference in its mean values extracted from 2 studies [21, 23], both of which compared this variable between two groups. The results showed a statistically significant difference favoring β-TCP in the SMD values with the fixed-effect but not with the random-effect criteria (t = 3.730, *p* = 0.001; t = 1.844, *p* = 0.075, respectively). Cochran’s Q value was not statistically significant (Q = 3.707, *p* = 0.0542), and the I^2^ value (73.02%) was high but not statistically significant, which implies the absence of heterogeneity in the two studies included in the analysis. Hence, the pooled SMD obtained by the fixed-effect criteria was used to infer a significant difference in the mean values of PD reduction between the two groups (SMD = 1.555, t = 3.730, *p* = 0.001). The overall effect (1.555) was large (Table 3). The corresponding forest plot for PD reduction shows the effect sizes of each of the two studies and the combined effect size by the fixed- and random effects models (Fig. 3a).

**Table 3:** Meta-analysis of PD reduction, CAL gain, and bone fill variables related to two-wall infra-bony defects

PD reduction	Group 1			Group 2			SMD	95% confidence interval (CI)	
Study	Total	Mean	SD	Total	Mean	SD			
Sturb JR et al., 1979	10	1.9	0.5	3	1.5	0.8	0.657	− 0.720, 2.034	
Rajesh et al., 2009	10	6.2	1.1	10	4.3	0.27	2.272	1.097, 3.446	
*Overall effect* Fixed effects: Total N = 33; SMD = 1.555 (95% CI: 0.705, 2.405); t = 3.730; *p* = 0.001 Random effects: Total N = 33; SMD = 1.489 (95% CI − 0.158, 3.135); t = 1.844; *p* = 0.075 Test for heterogeneity: Q = 3.707; *p* = 0.0542; I^2^ = 73.02% (95% CI 0.00%, 93.93%)								Weight (%)	
								Fixed	Random
								44.40 55.60	48.49 51.51

CAL gain	Group1			Group2			SMD	95% CI	
Study	Total	Mean	SD	Total	Mean	SD			
Rajesh et al., 2009	10	5.6	1.2	10	3.3	1.1	0.524	0.812, 3.015	
Zafiropoules et al., 2007	4	4.0	0.8	9	4.4	0.8	− 0.465	− 1.711, 0.781	
*Overall effect* Fixed effects: Total N = 33; SMD = 0.815 (95% CI 0.031, 1.60); t-value = 2.119; *p* = 0.042 Random effects: Total N = 33; SMD = 0.734 (95% CI − 1.692, 3.159); t-value = 0.617; *p* = 0.542 Test for heterogeneity: Q = 9.499; *p* = 0.002; I^2^ = 89.47% (95% CI 60.84%, 97.17%)								Weight (%)	
								Fixed	Random
								53.83 46.17	50.40 49.60

Bone fill	Group1			Group2			SMD	95% CI	
Study	Total	Mean	SD	Total	Mean	SD			
Sturb JR et al., 1979	10	1.2	0.6	3	2.4	1.3	0.674	− 2.922, 0.045	
Zafiropoules et al., 2007	4	6.5	1.7	9	7.7	0.8	0.592	− 2.30, 0.306	
*Overall effect* Fixed effects: Total N = 26; SMD = − 1.189 (95% CI − 2.107, − 0.271); t-value = − 2.673; *p* = 0.013 Random effects: Total N = 26; SMD = − 1.189 (95% CI − 2.107, − 0.271); t-value = − 2.673; *p* = 0.013 Test for heterogeneity: Q = 0.2425; *p* = 0.622; I^2^ = 0.00% (95% CI 0.00%, 0.00%)								Weight (%)	
								Fixed	Random
								43.55 56.45	43.55 56.45

*PD* pocket depth, *SMD* standardized mean difference, *SD* standard deviation, *CAL* clinical attachment level

For another outcome variable “CAL gain,” the results again showed a statistically significant difference favoring β-TCP in the SMD values obtained by the fixed-effect but not by the random-effect criteria (t = 2.119, *p* = 0.042; t = 0.617, *p* = 0.542, respectively). Cochran’s Q value was statistically significant (Q = 9.499, *p* = 0.002) and the I^2^ value (89.47%) was high and statistically significant, which indicated heterogeneity in the two studies included in the analysis. Thus, the pooled SMD by random-effect criteria was used to infer the absence of significant differences in the mean values of CAL gain between the two groups (SMD = 0.734, t = 0.617, *p* = 0.542). The overall effect (0.734) was medium (Table 3). The corresponding forest plot for CAL gain shows the effect sizes of each of the two studies and the combined effect size obtained by the fixed- and random effects models (Fig. 3b).

For the third outcome variable “BF,” the results showed a statistically significant difference from the control groups in the SMD values with both the fixed-effect and random-effect criteria (t = 2.673, *p* = 0.013; t = 2.673, *p* = 0.013, respectively). Cochran’s Q value was not statistically significant (Q = 0.2425, *p* = 0.622) and the I^2^ value was 0.00%, which implies the absence of heterogeneity in the two studies included in the analysis. Thus, the pooled SMD obtained by fixed-effect criteria was used to infer a significant difference in the mean values of BF between the two groups (SMD = 1.189, t = 2.673, *p* = 0.013). The overall effect (1.189) was large (Table 3). The corresponding forest plot for BF showed the effect sizes of each of the two studies and their combined effect size by the fixed- and random effects models (Fig. 3c) (Table 3).

#### Three-wall infra-bony defects

**Fig. 4:**
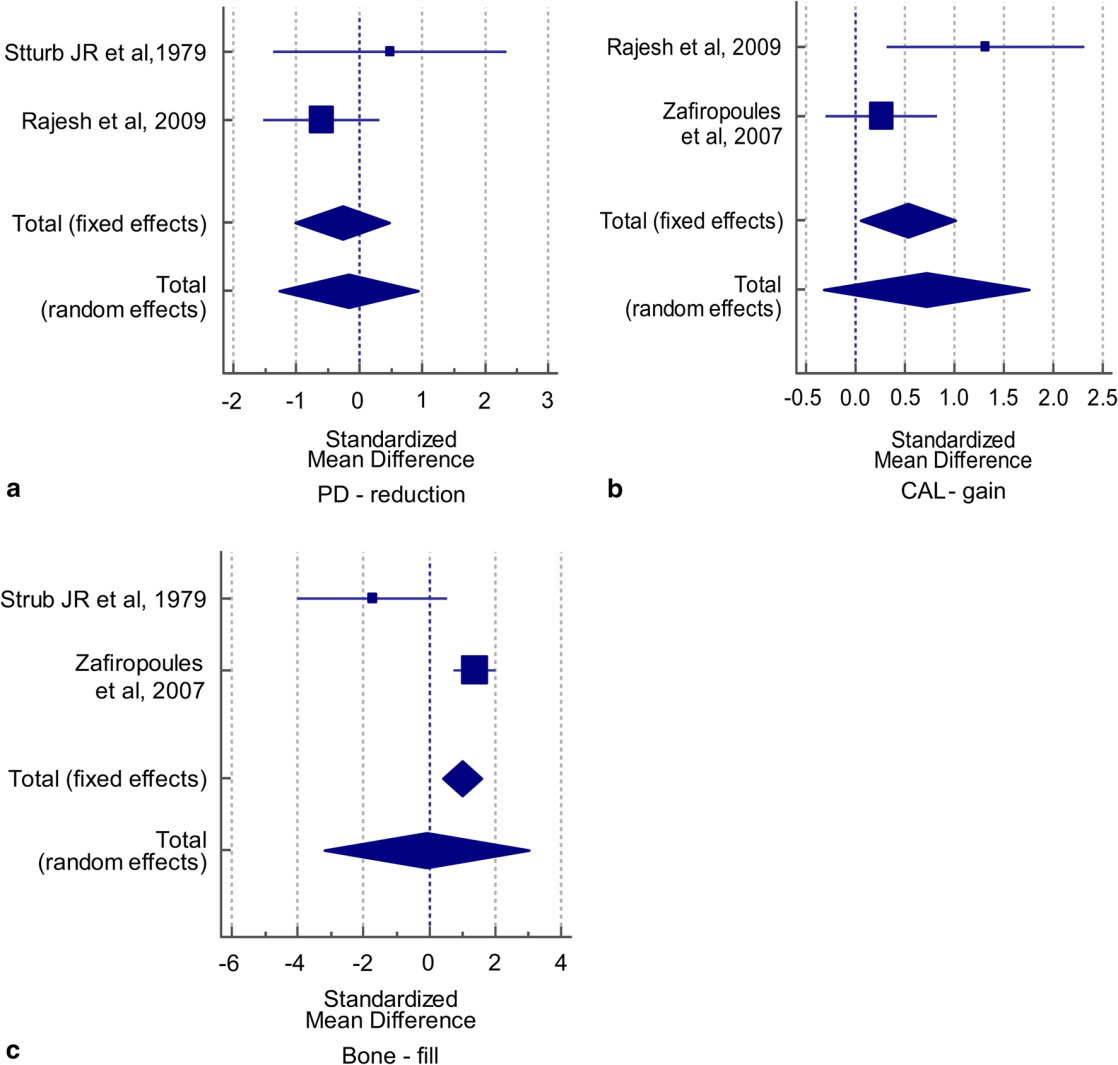
Forest plot for primary outcome variables of three-wall infra-bony walls defects

For the outcome variable “PD reduction,” the results showed no significant difference in the SMD values obtained by both fixed- and random-effect criteria (t = 0.744, *p* = 0.464; t = 0.322, *p* = 0.750, respectively). Cochran’s Q value was not statistically significant (Q = 1.873, *p* = 0.171) and the I^2^ value (46.61%) was low and not statistically significant, implying the absence of heterogeneity in the two studies included in the analysis. Thus, the pooled SMD obtained by fixed-effect criteria was used to infer the absence of significant differences in the mean values of PD reduction between the two groups (SMD = 0.273, t = 0.744, *p* = 0.464). The overall effect (0.273) was low (Table 4). The corresponding forest plot for PD reduction shows the effect sizes of each of the two studies and the combined effect size obtained by the fixed- and random effects models (Fig. 4a).

**Table 4:** Meta-analysis of PD reduction, CAL gain, and bone fill variables related to three-wall infra-bony defects

PD reduction	Group 1			Group 2			SMD	95% confidence interval (CI)	
Study	Total	Mean	SD	Total	Mean	SD			
Sturb JR et al., 1979	3	1.9	0.6	3	1.5	0.7	0.667	− 1.361, 2.340	
Rajesh et al., 2009	10	3.5	1.2	10	4.5	1.9	0.439	− 1.524, 0.319	
*Overall effect* Fixed effects: Total N = 26; SMD = − 0.273 (95% CI − 1.029, 0.484); t = − 0.744; *p* = 0.464 Random effects: Total N = 26; SMD = − 0.172 (95% CI − 1.274, 0.930); t = − 0.322; *p* = 0.750 Test for heterogeneity: Q = 1.873; *p* = 0.171; I^2^ = 46.61% (95% CI 0.00%, 0.00%)								Weight (%)	
								Fixed	Random
								30.22 69.78	39.44 60.56

CAL gain	Group 1			Group 2			SMD	95% CI	
Study	Total	Mean	SD	Total	Mean	SD			
Rajesh et al., 2009	10	5.9	1.2	10	3.8	1.8	1.315	0.315, 2.315	
Zafiropoules et al., 2007	25	4.7	0.8	25	4.5	0.7	0.262	− 0.300, 0.824	
*Overall effect* Fixed effects: Total N = 35; SMD = 0.532 (95% CI 0.051, 1.013); t-value = 2.206; *p* = 0.031 Random effects: Total N = 35; SMD = 0.718 (95% CI − 0.323, 1.759); t-value = 1.376; *p* = 0.173 Test for heterogeneity: Q = 3.636; *p* = 0.056; I^2^ = 72.50% (95% CI 0.00%, 93.81%)								Weight (%)	
								Fixed	Random
								25.65 74.35	43.30 56.70

Bone fill	Group 1			Group 2			SMD	95% CI	
Study	Total	Mean	SD	Total	Mean	SD			
Sturb JR et al., 1979	3	1.2	0.5	3	2.4	0.6	− 1.734	− 4.015, 0.547	
Zafiropoules et al., 2007	25	7.7	0.9	25	6.3	1.1	1.371	0.747, 1.995	
*Overall effect* Fixed effects: Total N = 56; SMD = 0.983 (95% CI 0.401, 1.565); t-value = 3.388; *p* = 0.001 Random effects: Total N = 56; SMD = − 0.088 (95% CI − 3.195, 3.019); t-value = − 0.057; *p* = 0.955 Test for heterogeneity: Q = 12.500; *p* = 0.0004; I^2^ = 92.00% (95% CI 72.42%, 97.68%)								Weight (%)	
								Fixed	Random
								12.49 87.51	47.00 53.00

*PD* pocket depth, *SMD* standardized mean difference, *SD* standard deviation, *CAL* clinical attachment level

For the outcome variable “CAL gain,” the results showed a statistically significant difference favoring β-TCP in the SMD values obtained by the fixed-effect but not by the random-effect criteria (t = 2.206, *p* = 0.031; t = 1.376, *p* = 0.173, respectively). Cochran’s Q value was not statistically significant (Q = 3.636, *p* = 0.056) and the I^2^ value (72.50%) was high, but not statistically significant, which implies that there was no heterogeneity in the two studies included in the analysis. Thus, the pooled SMD obtained by the fixed-effect criteria was used to indicate a significant difference in the mean values of CAL gain between the two groups (SMD = 0.532, t = 2.206, *p* = 0.031). The overall effect (0.532) was medium (Table 4). The corresponding forest plot for CAL gain shows the effect sizes of each of the two studies and the combined effect size obtained by the fixed- and random effects models (Fig. 4b).

For the outcome variable “BF,” the results showed a statistically significant difference favoring β-TCP in the SMD value with only the fixed-effect criteria but not with the random-effect criteria (t = 3.388, *p* = 0.001; t = 0.057, *p* = 0.955, respectively). Cochran’s Q value was highly statistically significant (Q = 12.50, *p* = 0.0004), and the I^2^ value (92.00%) implied high heterogeneity in the two studies that were included in the analysis. Thus, the pooled SMD obtained by the random-effect criteria was used to infer no statistically significant difference in the mean values of BF between the two groups (SMD = 0.088, t = 0.057, *p* = 0.955). The overall effect (0.088) was low (Table 4). The corresponding forest plot for BF shows the effect sizes of each of the two studies and the combined effect size by the fixed- and random effects models (Fig. 4c) (Table 4).

## Discussion

This review and meta-analysis was conducted to evaluate the periodontal regenerative outcomes of using β-TCP alloplast for treating infra-bony defects and to compare the findings with those obtained after using other grafting or regenerative materials. This extensive literature search revealed that very few studies had been performed on this alloplastic material. Only five studies were included in this systematic review, three of these had been classified as showing a low risk of heterogeneity and were involved in the meta-analysis. Overall, β-TCP demonstrated favorable results compared to debridement alone. However, using it alone or in combination with other bone substitutes showed outcomes comparable to those with other treatment modalities and grafting materials. Furthermore, the meta-analysis revealed that for two-wall defects, the use of β-TCP was associated with a significant difference in PD reduction and CAL gain; BF did not show such a change. However, for three-wall defects, while no statistically significant difference was observed in PD reduction with both random- and fixed-effect models, CAL gain and BF showed statistically significant differences favoring the use of β-TCP in the fixed-effect model. Overall, all outcome measures showed comparable results for β-TCP and all other treatment modalities and grafting materials used. The amount of PD reduction, CAL gain, and BF was slightly inferior to those with autogenous and allografts only, in comparison with the fixed-effect meta-analysis model. Similar results have been reported by Calin et al., who noted that the β-TCP group showed comparable PD reduction, CAL gain, and BF as autogenous and allogenous grafts [25]. In contrast, in a previous systematic review comparing the amount of BF using autografts, allografts, xenografts, and alloplasts, BF was slightly inferior in the alloplast groups, compared with the other groups [26].

Furthermore, we compared the outcomes of β-TCP in this review with those of another well-documented alloplastic material, HA. A recent systematic review showed no significant difference between the use of HA and open-flap debridement and concluded that the clinical effectiveness of HA in treating periodontal bony defect regeneration is unclear. However, the combination of HA with β-TCP showed significant improvement in bone defect regeneration [27].

Overall, the autografts and allografts showed superior results in terms of regeneration, especially with more challenging defects such as two-wall defects, as they were less predictable and required an additional focused approach to successful regenerative outcomes. β-TCP is a promising alternative regenerative material in situations where optimum grafts cannot be used due to unavailability or cost issues. Furthermore, it is possible to enhance β-TCP outcomes when combined with other types of alloplasts or growth factors to achieve similar outcomes to autografts and allografts [25–28].

This systematic review has several limitations, including the limited number of studies, lack of randomized clinical trials, and small sample sizes of the included studies.

## Conclusions

The findings of this systematic review and meta-analysis suggest that β-TCP is a promising alternative to bone substitute material when used in treating periodontal infra-bony defects around natural teeth, warranting further exploration and investigation in comparative assessments. Additional randomized controlled trials focusing on β-TCP are required to confirm the current findings.

## Data Availability

The datasets used and/or analyzed during the current study are available from the corresponding author on reasonable request.

## Abbreviations

β-TCP: β-Tricalcium phosphate
ASB: Autogenous spongiosa
CAL: Clinical attachment level
GR: Gingival recession
GTR: Guided tissue regeneration
HA: Hydroxyapatite
KTW: Keratinized tissue width
PD: Probing depth
STT: Soft tissue thickness
SMD: Standardized mean difference
